# Supplementary figures and images for: Rapid-Throughput Skeletal Phenotyping of 100 Knockout Mice Identifies 9 New Genes That Determine Bone Strength
Source: PLoS Genet. 2012 Aug 2;8(8):e1002858. doi: 10.1371/journal.pgen.1002858 (PMC3410859; doi:10.1371/journal.pgen.1002858)

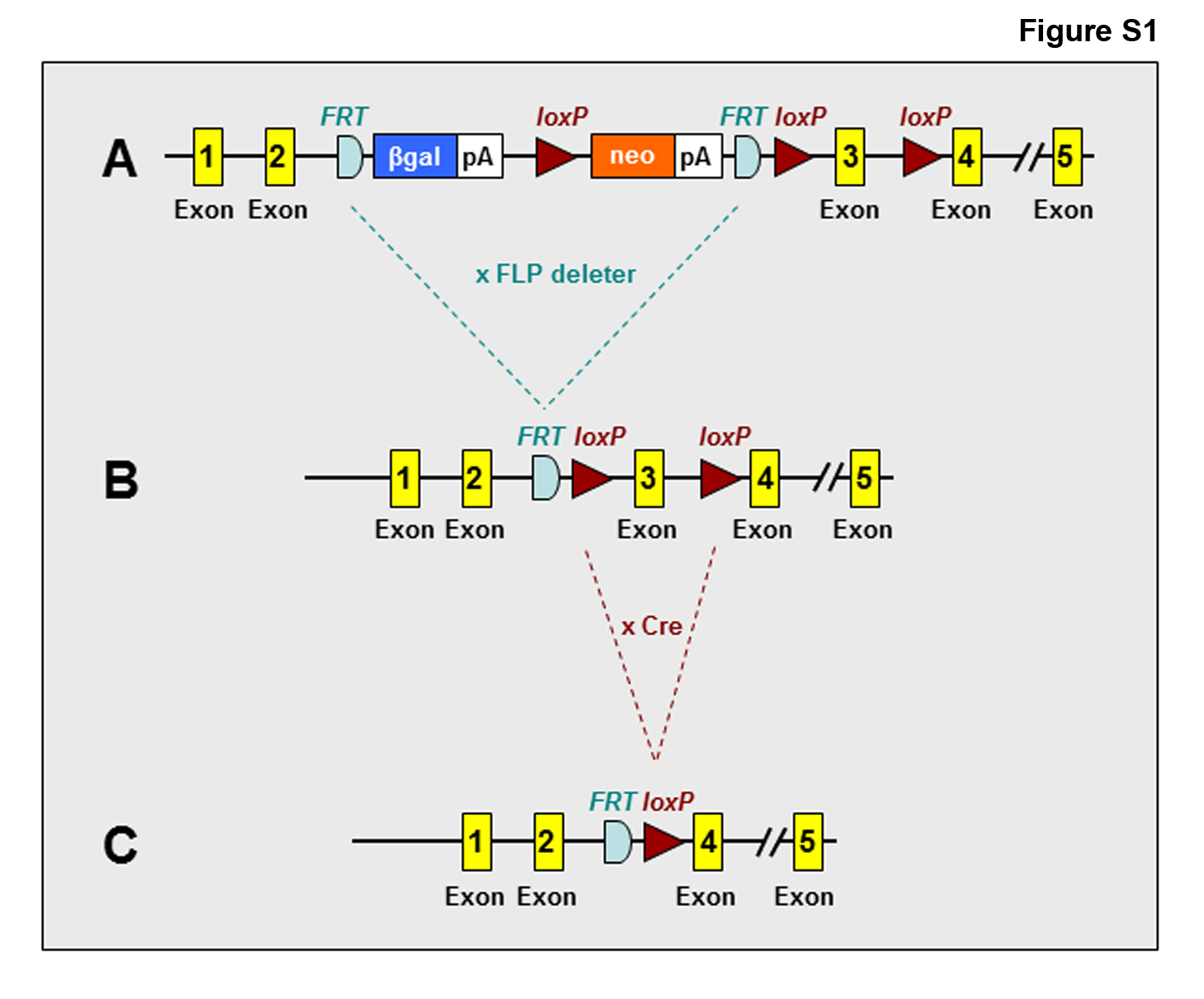

Supplement: Figure S1 — International knockout mouse consortium gene targeting strategy. A, Global gene inactivation is achieved using a gene-trap targeting cassette containing a LacZ gene expressing β-galactosidase and a neomycin marker gene flanked by Flp recombinase target sites (FRT), together with a critical exon flanked by loxP sites (exon 3 in this example). Gene expression from the targeted allele can be determined by X-gal staining for β-galactosidase activity. B, Crossing targeted mice with a Flp deleter strain removes the lacZ and neomycin genes, resulting in reversal of the gene-trap knockout and generation of a floxed allele. C, Mice harboring the floxed allele can be crossed with appropriate Cre recombinase expressing strains to generate tissue-specific knockout mice for further study. (TIF) [file pgen.1002858.s001.tif]

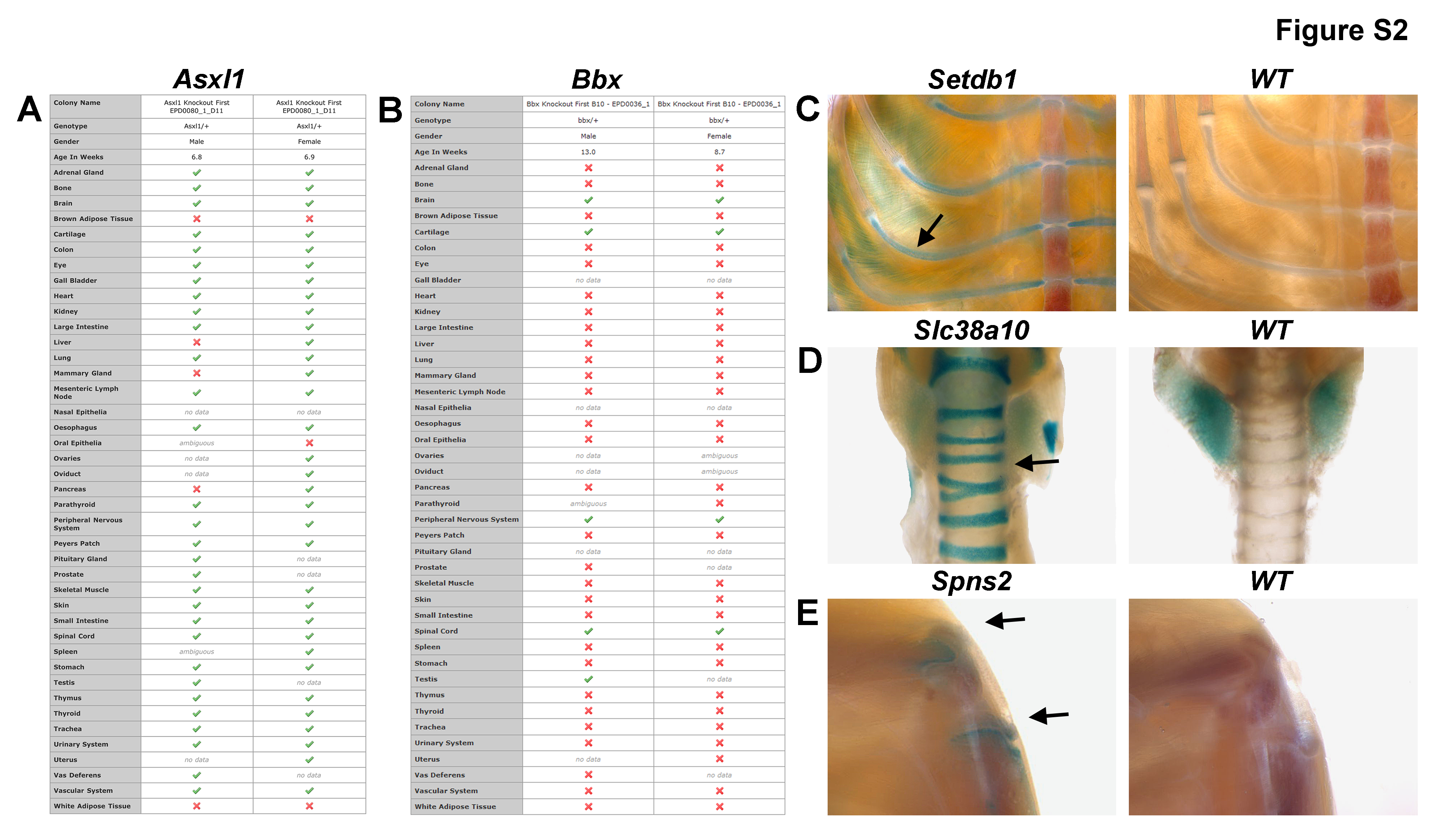

Supplement: Figure S2 — Determination of the tissue distribution of gene expression. A, Widespread tissue distribution of Asxl1 expression as determined by X-gal staining of tissue preparations for lacZ reporter gene expression in heterozygous mice. Table shows the annotations as reported on the Wellcome Trust Sanger Institute Mouse Genetics Project portal (http://www.sanger.ac.uk/mouseportal/). B, Restricted tissue expression pattern of Bbx. C, Expression of Setdb1 in rib cartilage (arrow), as demonstrated by X-gal staining (blue) of tissue preparation from a 7 week old heterozygote. D, Expression of Slc38a10 in tracheal cartilage (arrow) in an 8 week old heterozygote. E, Expression of Spns2in distal femur and proximal tibia (arrows) in an 8 week old heterozygote. Corresponding images from wild type (WT) control mice show background X-gal staining. (TIF) [file pgen.1002858.s002.tif]

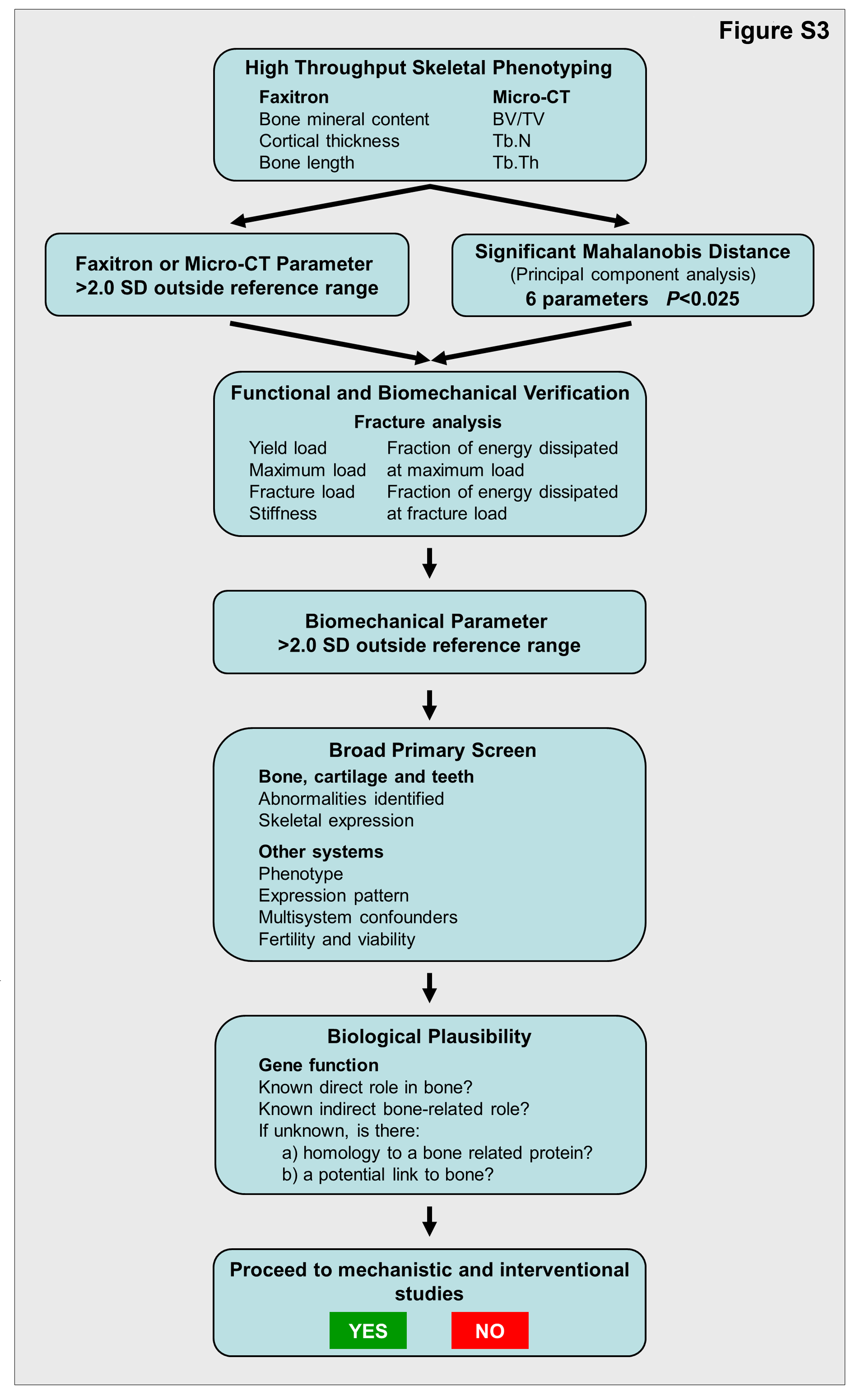

Supplement: Figure S3 — Multi-parameter and functional skeletal phenotyping algorithm. Summary of an unbiased and high-throughput phenotype screen to identify knockout mice with skeletal abnormalities and identify new genetic determinants of bone mass and strength. (TIF) [file pgen.1002858.s003.tif]

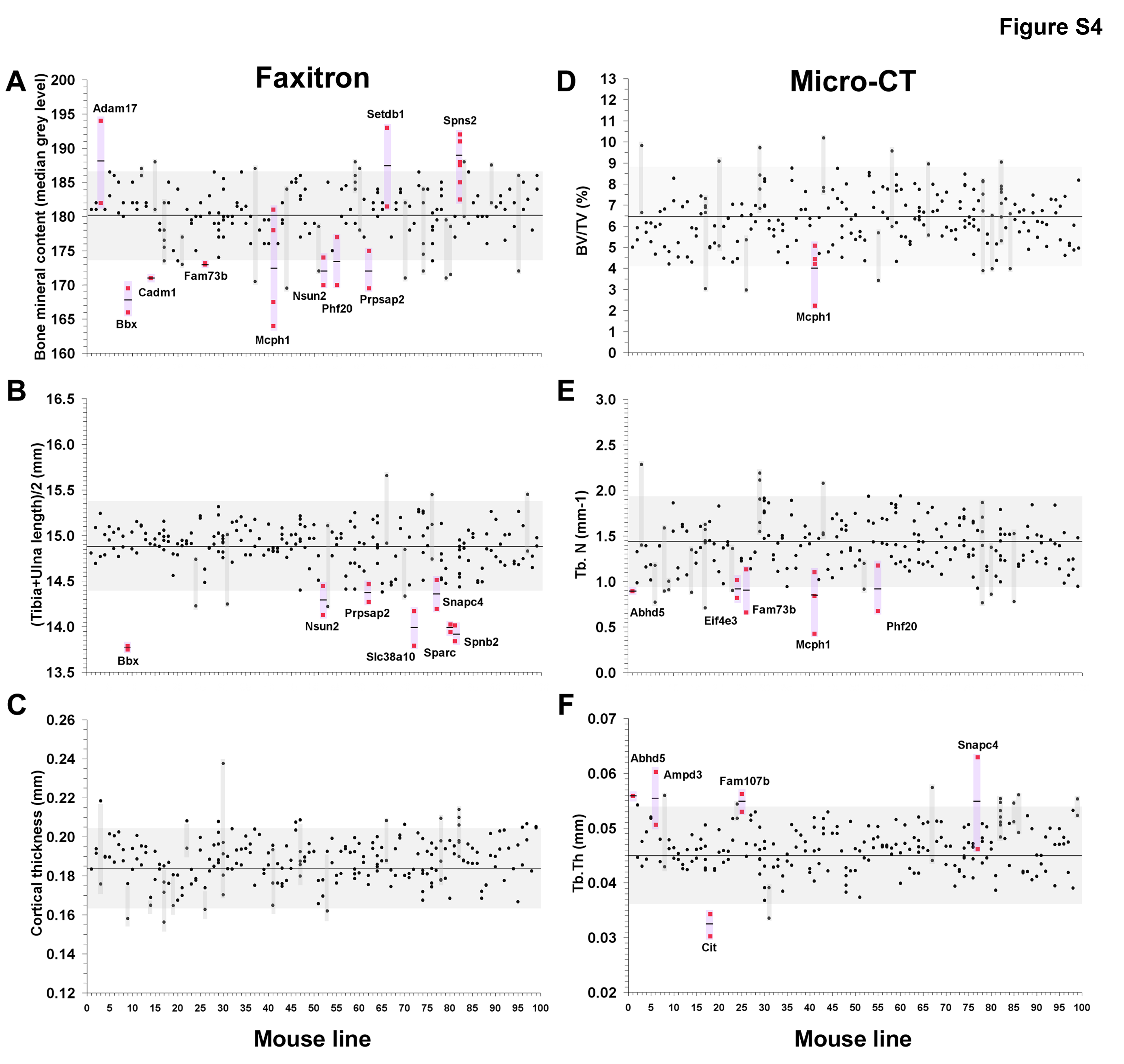

Supplement: Figure S4 — Bone structure determined by Faxitron x-ray microradiography and micro-CT in 100 unselected knockout mouse strains. The mean value for each parameter obtained from n = 77 female 16 week old WT mice is shown as a horizontal line with the 2.0 SD reference range limits in grey. Black dots represent values for individual knockout mice. Grey vertical boxes highlight the distributions of values from mice of a single strain in which at least one animal lies outside the reference range, but the mean value lies within the reference range. Violet vertical boxes and red squares highlight the distributions of individual values from outlier strains in which the mean value (horizontal line in violet box) lies outside the reference range. Gene symbols for outlier strains are indicated. A–C,</!emph> Faxitron x-ray microradiography measures of bone mineral content, bone length and cortical thickness. D–F, Micro-CT measures of bone volume/tissue volume (BV/TV), trabecular number (Tb.N) and trabecular thickness (Tb.Th). (TIF) [file pgen.1002858.s004.tif]

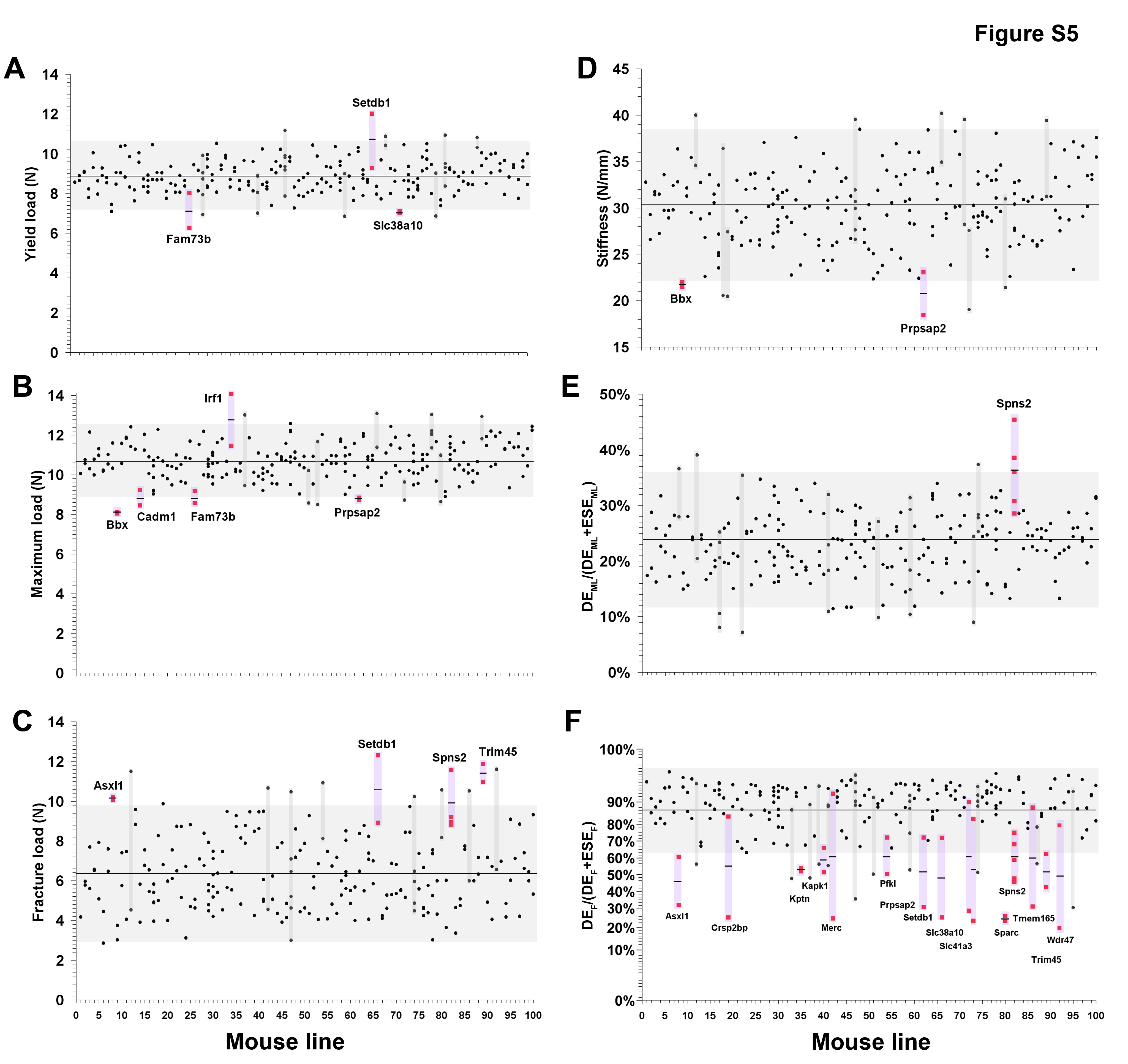

Supplement: Figure S5 — Bone strength determined by destruction 3-point bend testing in 100 unselected knockout mouse strains. The mean value for each parameter obtained from n = 77 female 16 week old WT mice is shown as a horizontal line with the 2.0SD reference range limits shaded in grey. Black dots represent values for individual knockout mice. Grey vertical boxes highlight the distributions of values from mice of a single strain in which at least one animal lies outside the reference range, but the mean value lies within the reference range. Violet vertical boxes and red squares highlight the distributions of individual values from outlier strains in which the mean value (horizontal line in violet box) lies outside the reference range. Gene symbols for outlier strains are indicated. A–F, Yield load, maximum load, fracture load, stiffness, the proportion of energy dissipated at maximum load, and the proportion of energy dissipated at fracture. (TIF) [file pgen.1002858.s005.tif]

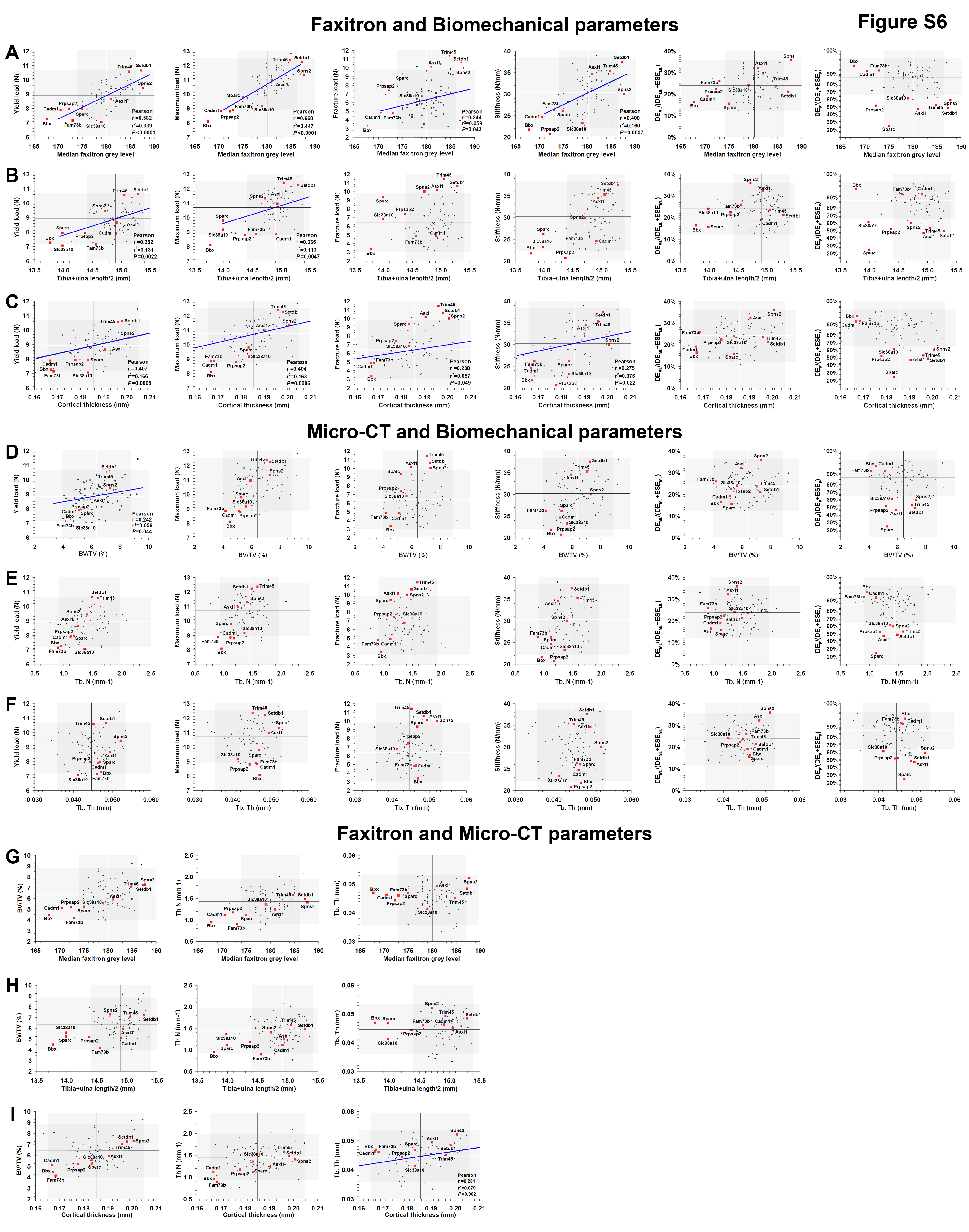

Supplement: Figure S6 — Relationship between bone structure and strength. In each graph individual wild-type mice are identified by black spots. The 10 strains with major phenotypes are identified by red squares and gene symbols. The 2.0SD reference range for each variable is represented by the grey box. Pearson correlation coefficients for significant relationships are indicated and blue lines demonstrate significant linear correlations. A–C, Biomechanical and Faxitron parameters. Scatter graphs showing each of the six biomechanical parameters (yield load, maximum load, fracture load, stiffness, proportion of energy dissipated at maximum load, proportion of energy dissipated at fracture) plotted versus each of the three structural parameters (median grey value, cortical thickness, bone length) obtained by Faxitron x-ray microradiography. D–F, Biomechanical and micro-CT parameters. Scatter graphs showing each of the six biomechanical parameters (yield load, maximum load, fracture load, stiffness, proportion of energy dissipated at maximum load, proportion of energy dissipated at fracture) plotted versus each of the three structural parameters (BV/TV, Tb. N, Tb. Th) obtained by micro-CT. G–I, Faxitron and micro-CT parameters. Scatter graphs showing each of the three structural parameters obtained by micro-CT versus each of the three structural parameters obtained by Faxitron x-ray microradiography. (TIF) [file pgen.1002858.s006.tif]
