# Supplementary material for: Rapid-Throughput Skeletal Phenotyping of 100 Knockout Mice Identifies 9 New Genes That Determine Bone Strength
Source: PLoS Genet. 2012 Aug 2;8(8):e1002858. doi: 10.1371/journal.pgen.1002858 (PMC3410859; doi:10.1371/journal.pgen.1002858)
Supplement: Table S2 — Accuracy of methods to identify major phenotypes affecting the structure and strength of bone. The sensitivities, specificities and positive and negative predictive values of x-ray microradiography, micro-CT, Mahalanobis calculations and broad primary phenotyping are shown. (DOC) [file pgen.1002858.s008.doc]

**Table S2**

|  | **Sensitivity** | **Specificity** | **Positive**  **Predictive Value** | **Negative Predictive Value** |
| --- | --- | --- | --- | --- |
| **Faxitron x-ray microradiography** | 80% | 93% | 57% | 98% |
| **Micro-CT** | 10% | 91% | 11% | 90% |
| **Mahalanobis** | 80% | 65% | 20% | 97% |
| **Faxitron, Micro-CT and Mahalanobis** | 100% | 63% | 23% | 100% |
| **Broad 1^0^ Screen** | 50% | 89% | 33% | 94% |
